# A Novel Thiophene-Fused Polycyclic Aromatic with a Tetracene Core: Synthesis, Characterization, Optical and Electrochemical Properties

**DOI:** 10.3390/molecules16064467

**Published:** 2011-05-27

**Authors:** Zong-Fan Duan, Xian-Qiang Huang, Zhi-Gang Yang, Daiki Hoshino, Susumu Kitanaka, Gao-Yang Zhao, Yasushiro Nishioka

**Affiliations:** 1 College of Science and Technology, Nihon University, Narashinodai, Funabashi, Chiba 2748501, Japan; 2 School of Materials Science and Engineering, Xi’an University of Technology, Xi’an 710048, China; 3 College of Chemistry and Chemical Engineering, Liaocheng University, Shandong 252059, China; 4 College of Pharmacy, Nihon University, Narashinodai, Funabashi, Chiba 2748555, Japan

**Keywords:** polycyclic aromatics, oxidative cyclization, organic semiconductor materials

## Abstract

FeCl_3_-mediated oxidative cyclization was successfully used to construct an extended thiophene-pendant pyrene skeleton and synthesize a novel thiophene-fused polycyclic aromatic (THTP-C) with a tetracene core. The identity of the compound was confirmed by ^1^H-NMR, ^13^C-NMR, MS, and elemental analysis. Meanwhile, a single crystal of THTP-C was obtained and analyzed by X-ray single-crystal diffraction. THTP-C has a “saddle” shaped π-conjugated 1-D supramolecular structure, and favors highly ordered self-assembly by π-π interactions as evidenced by its concentration-dependent ^1^H-NMR spectra in solution. The optical properties of THTP-C were investigated by ultraviolet-visible (UV-Vis) and photoluminescence (PL) spectroscopy and its electrochemical properties were investigated by cyclic voltammetry (CV). The relatively large band gap (2.86 eV), low E_HOMO_ level (−5.64 eV) and intermolecular π-π interactions imply that THTP-C has a high stability against photo-degradation and oxidation, and may be a promising candidate for stable hole-transporting materials.

## 1. Introduction

Organic semiconductors based on π-conjugated aromatics have been well-investigated due to their potential applications as active organic semiconductor layers in a variety of organic electronic devices. Acene and oligothiophene derivatives with high charge-carrier mobility in the solid state represent two of the most heavily studied series of compounds and are used as organic semiconductor materials in organic light emitting diodes (OLEDs), solar cells and organic field effect transistors (OFETs) [1,2]. Many efforts have been devoted to the synthesis and applications in organic-based electronic devices of various π-conjugated homoaromatic and heteroaromatic systems, and oligomers and polymers derived from acene and thiophene derivatives. In particular, heteroaromatic oligomers derived from acene and thiophene have received considerable attention due to their unique photophysical properties and the feasibility of chemical modification, as well as their good stability against photo-degradation and oxidation. Thiophene-acene co-oligomers and thiophene-fused acenes are very attractive for maximizing π-orbital overlap by reducing the freedom of rotation in the oligomers and possibly inducing a densely packed crystal structure with face-to-face π-stacking motifs. Recently, divinyl- benzene, naphthalene, anthracene, tetracene and fluorene, *etc.*, have been successfully introduced into oligothiophene via co-oligomerization and were used as excellent stable semiconductor materials with relatively high band gaps and low levels of the highest occupied molecular orbital energy (E_HOMO_) [3,4,5,6,7]. However, reports on the synthesis and properties of thiophene-fused acenes are rather limited.

**Scheme 1:**
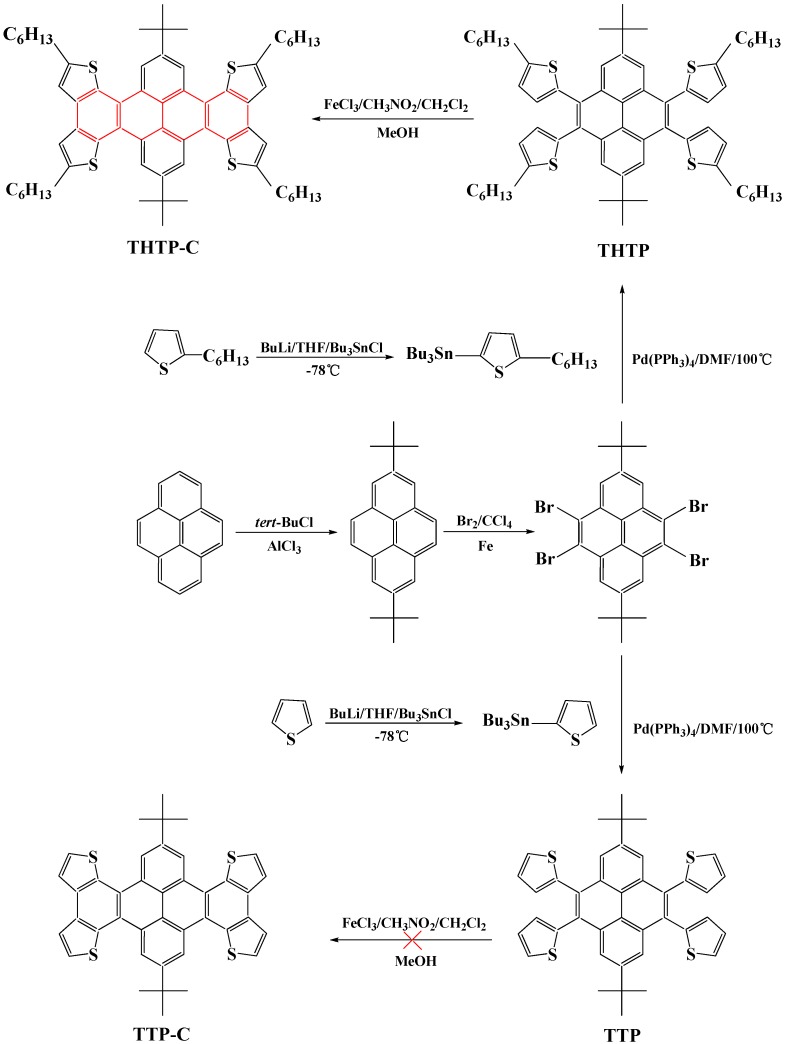
Synthetic pathway from pyrene to THTP-C.

Pyrene and its derivatives are well known as polycyclic aromatic hydrocarbons, and are mainly used as electro-conductive and photo-sensitive materials [8,9,10]. We have reported the successful synthesis of two new thiophene-containing oligomers based on a pyrene core with pendant thienyl rings [11]; an expanded and elongated conjugated system would be expected to lead to even more interesting electronic and optical properties. In this paper, FeCl_3_-mediated oxidative cyclization of 4,5,9,10-tetra[2-(5-hexyl)thienyl]-2,7-di-*tert*-butylpyrene (THTP) was used to construct an extended thiophene-pendant pyrene skeleton via thienyl-thienyl carbon-carbon bond formation and to eventually obtain the flat molecule THTP-C with a larger rigid tetracene core (Scheme 1). To assess its intrinsic potential as an opto-electronic and organic semiconductor material, the photophysical properties of THTP-C were studied using UV-Vis absorption and PL spectroscopy both in solution and thin film. The electrochemical properties were investigated using cyclic voltammetry (CV), and the respective energy levels were determined by a combinational analysis of CV with UV-Vis absorption. Finally, single-crystal crystallographic analysis was applied to elucidate the crystal structure of THTP-C.

## 2. Results and Discussion

### 2.1. Synthesis

Scheme 1 outlines the synthetic pathways to prepare THTP, TTP, and THTP-C. The facile synthetic approach for pyrene derivatives THTP and TTP with pendant thienyl rings mainly involved Friedel-Crafts alkylation, bromination, and Stille coupling reactions and was performed [11].

As an effective way to construct polycyclic aromatic hydrocarbons, FeCl_3_-mediated oxidative cyclization was utilized to construct an extended thiophene-fused oligoacene skeleton *via* thienyl-thienyl carbon-carbon bond formation [12,13,14,15,16]. The oxidative cyclization of THTP using various equivalents of FeCl_3_ was performed at different temperatures; the process was monitored using thin-layer chromatography (TLC). The selective β-β oxidative cyclization of THTP with 8 equiv. of FeCl_3_ carried out at 0 °C for 30 min resulted in the highest yield (78%) of THTP-C. The compound TTP, which has the pendant 2-thienyl rings without blocking groups on the reactive α- and β-sites, was also used for oxidative cyclization. It was envisioned that the β-β-sites of TTP would participate in discrete monomer cyclization to selectively afford TTP-C. Since both the α- and β-sites of the pendant monomer might undergo cyclization under oxidative conditions, and the α-sites were more reactive than the β-sites of pendant monomer, the oxidative cyclodehydrogenation of TTP gave a complicated mixture rather than the desired β-β oxidative cyclization compound TTP-C even if a small amount of FeCl_3_ (1.8 equiv. per formed C–C bond) and diluted concentrations were used. It was demonstrated by Tovar and Pei *et al.* that FeCl_3_-mediated cyclization usually could not be applied to electron-rich precursors because the higher spin density and electron-rich properties of the α-position of the thiophene ring favored C–C coupling reactions at the *α*-sites forming cross-linked oligomers and polymers rather than discrete monomer cyclization compounds. Therefore, the oxidative cyclization of the β-sites of the pendant monomers only occurred after protection of the thiophene α-positions [12,13,14].

### 2.2. NMR Spectroscopy

The structure of THTP-C was identified by ^1^H- and ^13^C-NMR spectroscopy, fast-atom-bombardment-mass spectrometry (FAB-MS) and elemental analysis. After cyclization, the resonance at 6.81 ppm, which was assigned to the β-H on the thiophene rings closer to the pyrene core in THTP, disappeared and the doublet at 6.71 ppm, which was assigned to the other β-H of thiophene rings, now appeared as a singlet with a significant 0.87 ppm downfield shift in the ^1^H-NMR spectrum of THTP-C. Furthermore, the exact mass decreased from 978.6 to 974.3, which corresponded to the exact loss of four hydrogen atoms from THTP to form THTP-C. These changes clearly indicate that four β-H atoms of THTP participated in oxidative cyclodehydrogenation to form a large aromatic π-system.

**Figure 1:**
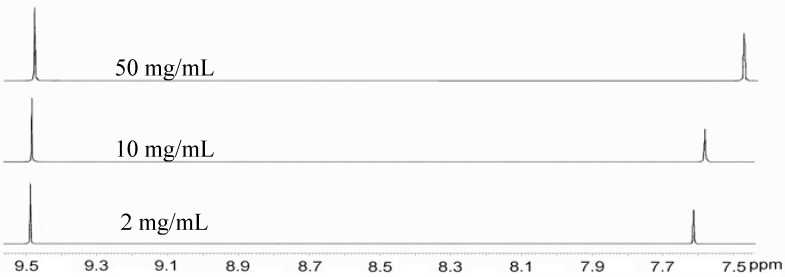
Comparison of the^1^H-NMR spectra of THTP-C in CDCl_3_ at different concentrations.

The highly extended π-framework contributes to effective molecular overlap, which can lead to self-association through π-π stacking between molecules. THTP-C favored highly ordered self-assembly by π-π interactions, which was evidenced by its concentration-dependent ^1^H-NMR spectra in solution. It was observed that the singlet at a chemical shift of 7.61 ppm, which is assigned to the protons in the fused-thiophene rings, significantly shifted to 7.47 ppm when the concentration of the CDCl_3_ solution increased from 2 to 50 mg/mL at room temperature, as shown in Figure 1. The upfield shift of the signals for these protons is attributed to intermolecular shielding from the fused-thiophene rings of neighboring aromatic molecules in the concentrated solution, which implies their tendency to self-associate through intermolecular π-π stacking [15,16]. However, no pronounced upfield shift was observed for the protons in the rings of pyrene core. The above results indicate that THTP-C has a tendency to self-associate through intermolecular π-π stacking, but the packing does not occur in well-defined columns, which was confirmed by X-ray crystallographic studies. In contrast, the other two compounds, THTP and TTP, showed no concentration-dependent chemical shift likely due to their small π-conjugated systems and the intermolecular steric repulsion between the twisted thiophene arms.

### 2.3. Photophysical Properties

**Figure 2:**
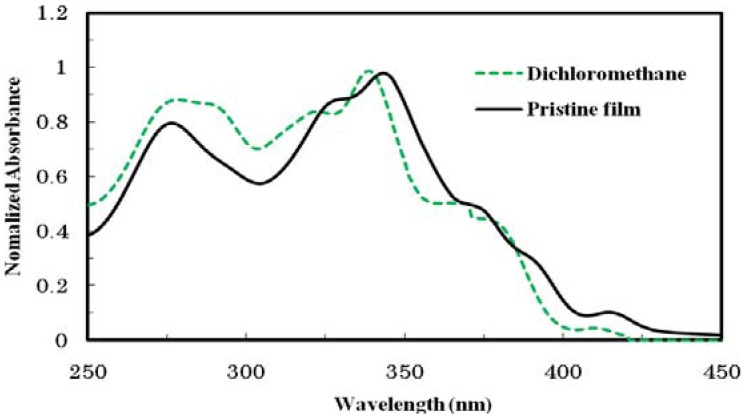
Normalized UV-Vis absorption spectra of THTP-C in dichloromethane and in a thin film.

The UV-vis absorption spectra of THTP-C in a CH_2_Cl_2_ solution (10^−5^ mol/L) and in a thin film fabricated by vacuum evaporation on quartz glasses are shown in Figure 2. The photophysical data for THTP-C, THTP, and TTP are summarized in Table 1.

**Table 1:** Optical and electrochemical data in thin films.

Compounds	UV-vis absorption		E_g_^opt^ (eV)	PL	Electrochemical data		
	λ_max_ (nm)	λ_edge_ (nm)		λ_max_ (nm)	E_onset_ (V)	E_HOMO_ (eV)	E_LUMO_ (eV)
TTP	359	386	3.21	426	1.12	−5.84	−2.63
THTP	362	400	3.10	412	0.96	−5.68	−2.58
THTP-C	344	433	2.86	443	0.92	−5.64	−2.78

**Figure 3:**
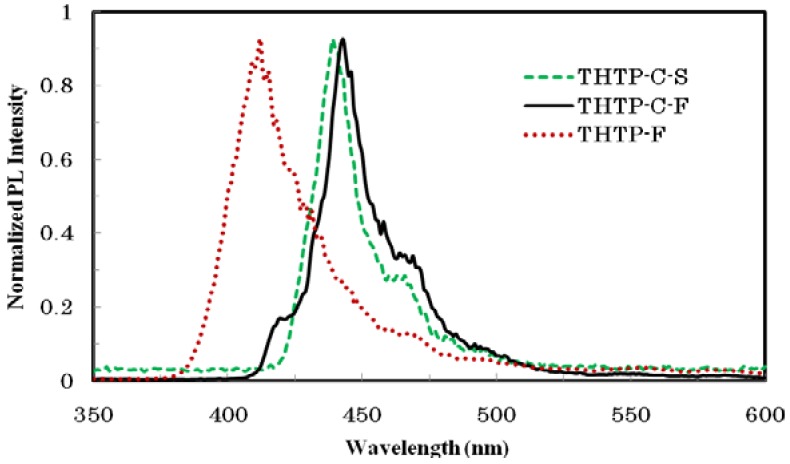
Normalized photoluminescence (PL) spectra: THTP-C in chloroform (THTP-C-S), THTP-C in film (THTP-C-F) and THTP in film (THTP-F).

As shown in Figure 2, two broad peaks were observed for THTP-C in solution and in a thin film. The one at a longer wavelength was around 320−350 nm and is attributed to the π-π* transition of the conjugated backbone, while the band at a shorter wavelength (λ_max_ = 250−300 nm) originated from the electronic transitions of the individual aromatic units. The absorption of THTP-C in dilute CH_2_Cl_2_ solution showed a maximum at 338 nm with a small peak at 410 nm. Compared with that in solution, the absorption spectrum of THTP-C in a thin film was less structured. The maximum absorption red-shifted by about 6 nm from 338 to 344 nm, while the small peak at 410 nm red-shifted about 5 nm to 415 nm; this was accompanied with obvious absorbance enhancement and a 13 nm red-shift in the absorption edge. The pronounced changes in the absorption spectra were a result of the delocalization of the exciton within a co-facial stack induced by the π-π interactions, which was also evidenced by a related red-shift of the photoluminescence (PL) spectra shown in Figure 3. The emission spectrum of THTP-C in dilute solution showed a maximum emission peak at 443 nm with board shoulder peaks at about 465 nm. The emission spectrum of THTP-C in a thin solid film had a similar structure to that in solution except that it was red-shifted about 5 nm. As illustrated in Table 1, in comparison with THTP, the maximum emission peak of THTP-C was red-shifted by more than 30 nm. This further indicates that a more delocalized and extended π-conjugated system was formed after cyclization. The optical band gap of THTP-C determined from the absorption onset [E_g_^opt^ (eV) = 1240 / λ_edge_ (nm)] was found to be 2.86 eV. This band gap is significantly higher than that of pentacene (2.2 eV), which is the most well-known OFET material, implying a greater stability of THTP-C against photo-degradation [5]. Compared to the optical band gaps of the un-fused compounds, THTP and TTP, a more than 0.2 eV decrease for THTP-C was observed. This slight decrease of optical band gap, which is usually observed in conjugated oligomers and polymers, also agrees with the formation of a more delocalized and extended π-conjugated system.

### 2.4. Electrochemical Properties

**Figure 4:**
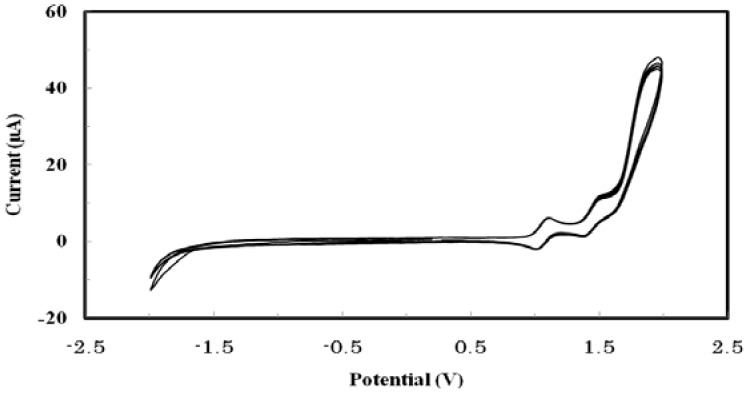
Cyclic voltammogram of THTP-C *vs.* Ag/AgCl electrode.

To understand the charge transport properties and assess the ionization potentials as well as the electrochemical stability of the oligomer, the redox properties of THTP-C (measured at a concentration of 10^−3^ M) were investigated by cyclic voltammetry. The analyte was dissolved in dichloromethane containing 0.1 M of tetrabutylammonium hexafluorophosphate (Bu_4_NPF_6_) as the supporting electrolyte and was analyzed at room temperature at a scan rate of 50 mV/s. A platinum stick electrode and platinum wire were used as the working electrode and counter electrode, respectively. An Ag/AgCl electrode was used as the reference electrode to calibrate against ferrocene/ferrocenium (0.08 V *vs.* Ag/AgCl) at the beginning of the experiments. The cyclic voltammograms of THTP-C are shown in Figure 4, and the onset oxidation potential and energy levels of the highest occupied molecular orbital (HOMO) and lowest unoccupied molecular orbital (LUMO) are summarized in Table 1. As shown in Figure 4, after five scans of THTP-C, only slight changes in both the potentials and current intensities were observed, demonstrating that THTP-C was stable to cycling through reduction and oxidation. THTP-C showed two quasi-reversible oxidation peaks and had an onset oxidation potentials (E_ox_) of 0.92 V (*vs*. Ag/AgCl). Assuming that the energy level of the ferrocene/ferrocenium (Fc) reference is 4.8 eV below vacuum, the energy level of HOMO (E_HOMO_) can be readily estimated from the onset oxidation potentials in the cyclic voltammogram [17]. Using the equation E_HOMO_ = −e(E_ox_ + 4.72) (eV), the HOMO energy level of THTP-C was calculated to −5.64 eV. The stability of organic semiconducting materials toward oxidative doping is related to their ionization potentials, *i.e.*, their HOMO energy levels from vacuum. Therefore, lowering the HOMO energy level would improve the environmental stability by minimizing the level of p-doping by ambient oxygen. Compared to that of pentacene (−4.96 eV) and other well-known current OFET materials [18], the lower HOMO energy level of THTP-C suggests that it should have better oxidative stability than traditional p-type semiconductor materials because there would be less probability of oxygen doping [7]. It is worth noting that such an energy level provides a closer match to the work function of platinum (5.6 eV) for source and drain contacts, which clearly encourages the preparation of prototype p-type OFET devices based on this material [19]. The LUMO energy level of THTP-C was estimated from the optical band gap and the HOMO energy value [17]. The calculated LUMO energy level of THTP-C is −2.78 eV. This LUMO energy level is higher than those of THTP and TTP, and matches well with the work function of Ca/Al (−2.76 eV) [20].

### 2.5. X-ray Crystallographic Analysis

**Figure 5:**
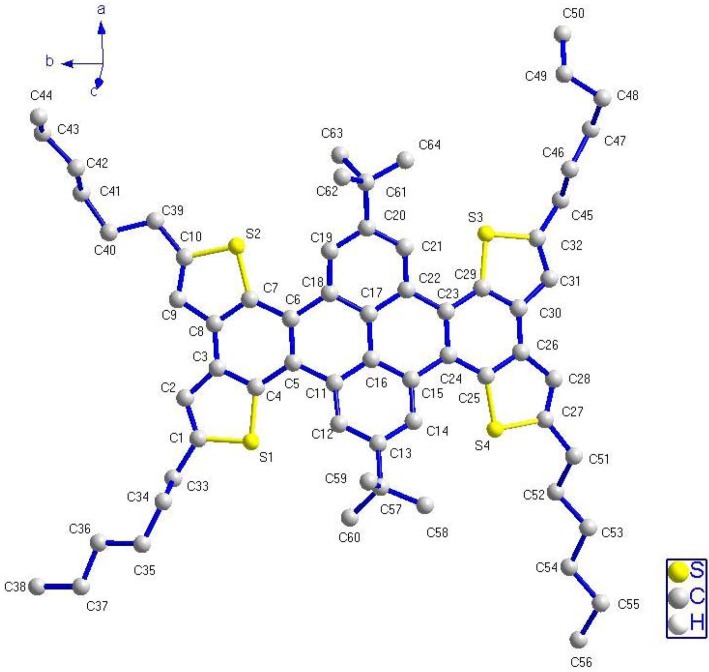
View of THTP-C showing the atomic numbering. Hydrogen atoms have been omitted for clarity.

**Figure 6:**
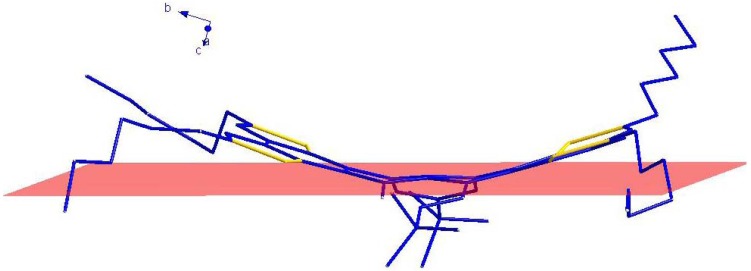
Model of “saddle” shaped THTP-C. An extension of the central plane formed by C22/C15/C11/C18 is shown in red.

**Figure 7:**
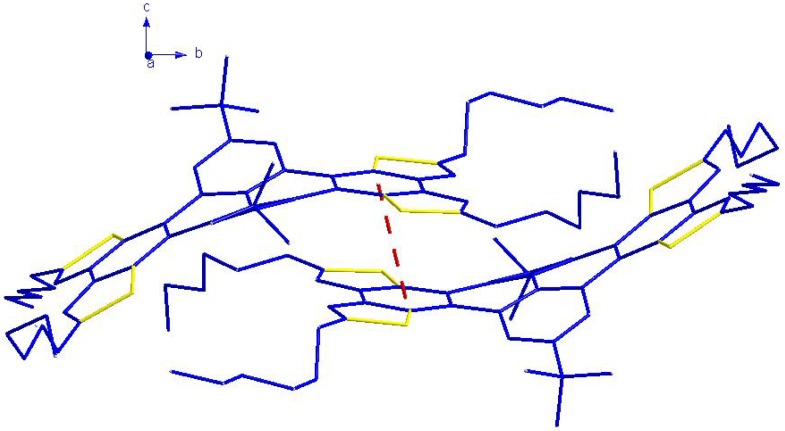
The π-π weak interactions between neighboring molecules (represented by red dashed lines).

**Figure 8:**
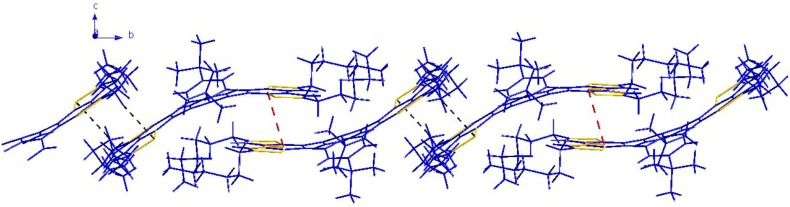
View of the one-dimensional supramolecular framework of THTP-C along the b axis. The π-π and CH-S interactions are represented by dashed red and black lines, respectively.

To further understand the molecular packing of THTP-C, a single crystal of THTP-C was grown by slow evaporation of solvent from its benzene solution, and the crystal structure was determined by X-ray diffraction. Crystallographic data were collected and analyzed. The basic information pertaining to the crystal parameters and structure refinement is summarized in Table 2, and selected bond lengths and angles are listed in Table 3. The single-crystal view and packing structure of THTP-C are shown in Figure 5, Figure 6 and Figure 7, Figure 8, respectively.

Contrary to our expectations, the new thiophene-fused acene was not planar and the observed deformation is, to the best of our knowledge, new to the thiophene-acene oligomer family [3]. With fused cores, we rarely observe a twist deformation as in acenes, but rather a curvature as in “saddle” shaped systems (Figure 6). The fused core and, therefore, the extended *π*-conjugated system of THTP-C are not planar, the tetracene core of THTP-C exhibits a upward curvature angle between the central two phenyl rings (C5/C6/C11/C16-C18, C15-C17/C22-C24) and two benzene rings’ planes (C3-C8, C23-C26/C30/C29) beside the central phenyl rings. The dihedral angle between the C22/C15/C11/C18 plane and the benzene ring (C3-C8) plane is 19.06(24)°; the C22/C15/C11/C18 plane and the benzene ring (C23-C26/C30/C29) plane also form a dihedral angle of 15.74(31)°, which may be due to the different steric configuration of the substituted alkyl groups. The main difference is the length of the bridging double bond, which decreases from 1.479(8) to 1.387(8) Å, which is indicative of compression and a weakly aromatic character of the central flat rings of THTP-C. Additionally, the central two phenyl planes (C5/C6/C11/C16-C18, C15-C17/C22-C24) and the other planes of two neighboring benzene rings (C11-C16, C17-C22) also formed a downward curvature angle. In this context, the overall molecular shape of THTP-C can be approximated by a “saddle” shaped systems. It is different from planar tetrathienoanthracene isomers without neighboring benzene rings reported by Brusso *et al.* [13]. The main factor that makes THTP-C prefer to “saddle” conformation must be the steric strain between planes of benzene rings. The steric strain between tetracene core and neighboring benzene rings’ planes (C11-C16, C17-C22) make tetracene core upward and neighboring planes downward, which result in the formation of “saddle” shaped conformation. Another factor is possibly due to the carbon-rich polyaromatic property of THTP-C, which makes THTP-C take on a “saddle” conformation to maintain the C–C bonds, just as carbon-rich polyaromatic molecules, such as fullerenes and fullerene fragments or buckybowls are inclined to form ball and bowl-shaped conformation to stabilize the molecular structure [21].

**Table 2:** Crystal data and structure refinement information for THTP-C.

Emprical formula	C_64_H_7__8_S_4_
Formula weight	975.50
Space group	C2/c
a [Å]	33.829(3)
b [Å]	19.9281(17)
c [Å]	18.3660(15)
α [˚]	90.00
β [˚]	113.6410(10)
γ [˚]	90.00
V [Å^3^]	11342.4(16)
Z	8
Dcalcd. [g cm^−3^]	1.143
θ range	2.38-25.02
index ranges	−32 ≤ h ≤ 40 − 23≤ k ≤ 18 – 20 ≤ l ≤ 21
R1, wR2^∗^ [I > 2σ(I)]	0.0889; 0.3567
GOF	1.073

^∗^ R1 = ∑||*F_o_*| − |*F_c_*||/|*F_o_*|. wR2 = [∑*w*(∑*F_o_*^2^ − *F_c_*^2^)^2^/∑*w*(*F_o_*^2^)^2^]^1/2^.

**Table 3:** Selected bond lengths (Å) and angles (˚) of the title compound.

S(1)-C(1)	1.734(7)	C(5)-C(11)	1.479(8)
S(1)-C(4)	1.737(6)	C(6)-C(7)	1.452(8)
S(2)-C(7)	1.734(6)	C(6)-C(18)	1.468(8)
S(2)-C(10)	1.736(8)	C(7)-C(8)	1.394(8)
S(3)-C(32)	1.733(7)	C(8)-C(9)	1.422(9)
S(3)-C(29)	1.745(7)	C(9)-C(10)	1.355(10)
S(4)-C(25)	1.734(7)	C(10)-C(39)	1.507(11)
S(4)-C(27)	1.744(7)	C(11)-C(12)	1.380(8)
C(1)-C(2)	1.346(9)	C(11)-C(16)	1.407(8)
C(1)-C(33)	1.504(8)	C(12)-C(13)	1.387(8)
C(2)-C(3)	1.425(8)	C(13)-C(14)	1.388(8)
C(2)-H(2)	0.9300	C(13)-C(57)	1.539(9)
C(3)-C(4)	1.388(8)	C(14)-C(15)	1.395(8)
C(3)-C(8)	1.409(8)	C(15)-C(16)	1.413(8)
C(4)-C(5)	1.436(8)	C(15)-C(24)	1.471(8)
C(5)-C(6)	1.395(8)	C(16)-C(17)	1.433(8)
C(18)-C(19)	1.387(8)	C(17)-C(22)	1.422(8)
C(19)-C(20)	1.384(8)	C(17)-C(18)	1.422(8)
C(20)-C(21)	1.389(9)	C(23)-C(24)	1.393(8)
C(21)-C(22)	1.388(8)	C(23)-C(29)	1.444(8)
C(22)-C(23)	1.478(8)	C(24)-C(25)	1.438(8)
C(1)-S(1)-C(4)	92.3(3)	C(4)-C(3)-C(8)	119.2(5)
C(7)-S(2)-C(10)	92.5(3)	C(4)-C(3)-C(2)	112.6(6)
C(32)-S(3)-C(29)	92.9(3)	C(8)-C(3)-C(2)	128.2(6)
C(25)-S(4)-C(27)	92.0(3)	C(3)-C(4)-C(5)	121.8(5)
C(2)-C(1)-C(33)	129.2(6)	C(3)-C(4)-S(1)	110.0(4)
C(2)-C(1)-S(1)	111.3(5)	C(5)-C(4)-S(1)	128.1(4)
C(33)-C(1)-S(1)	119.5(5)	C(6)-C(5)-C(4)	119.5(5)
C(1)-C(2)-C(3)	113.8(6)	C(6)-C(5)-C(11)	119.0(5)
C(1)-C(2)-H(2)	123.1	C(4)-C(5)-C(11)	121.5(5)
C(8)-C(7)-S(2)	109.6(5)	C(5)-C(6)-C(7)	118.0(5)
C(6)-C(7)-S(2)	129.1(5)	C(5)-C(6)-C(18)	120.1(5)
C(7)-C(8)-C(3)	120.1(5)	C(7)-C(6)-C(18)	121.8(5)
C(7)-C(8)-C(9)	113.6(6)	C(8)-C(7)-C(6)	121.3(5)
C(3)-C(8)-C(9)	126.3(6)	C(9)-C(10)-C(39)	129.9(8)
C(10)-C(9)-C(8)	112.9(6)	C(9)-C(10)-S(2)	111.5(5)
C(12)-C(13)-C(14)	117.2(5)	C(39)-C(10)-S(2)	118.5(7)
C(12)-C(13)-C(57)	119.7(5)	C(12)-C(11)-C(16)	118.7(5)
C(14)-C(13)-C(57)	123.0(5)	C(12)-C(11)-C(5)	123.4(5)

It is well known that the crystalline state can be controlled by considering size, shape, and aromatic interactions (both edge-to-face and face-to-face). The herringbone packing motif of unsubstituted pentacene represents a simple combination of edge-to-face and face-to-face interactions. In order to increase the π-stacking of pentacene by directed functionalization, Anthony reported a series of 6,13-disubstituted pentacenes in which the substituents were functionalized by ethyne units; the only strong interactions left to influence the solid-state order were aromatic face-to-face interactions [22]. Similar to the case of 6,13-bis(trimethylsilylethynyl)pentacene, the bulky *t*-Bu groups of THTP-C improve the quasi face-to-face interactions and discourage edge-to-face molecular interactions. As shown in Figure 7, it is clear that each THTP-C molecule is in close contact with the neighboring THTP-C molecules by two types of weak interactions: (1) The adjacent two molecules are packed into dimers through π-π stacking interactions of the benzene rings (C3-C8) at distance of 3.7395(2) Å; (2) CH–S interactions between the –CH moiety on the methylene group (C51) with the S atoms on the other thiophene rings of the THTP-C molecules at distances of 2.896(2) Å. All of these weak interactions play important roles in the formation of the 1-D structure of THTP-C (Figure 8); the 1-D structure is desirable for fast charge transport along the stacking axis and, thus, THTP-C could potentially serves as semiconductor material for electronic devices such as organic field effect transistors [15].

The compounds TTP and THTP are found to easily form large wire-shaped microstructures via a simple solution evaporation process, but attempts to grow single crystals suitable for X-ray crystallography have so far been unsuccessful likely as a result of their small π-conjugated systems and the intermolecular steric repulsion between the twisted thiophene arms.

## 3. Experimental

### 3.1. General

All commercially available chemicals were obtained from Tokyo Kasei Industry or Sigma-Aldrich and used as received. All solvents were of at least reagent grade. Tetrahydrofuran and diethyl ether were dried by distillation from sodium. *N*,*N*-dimethylformamide and dichloromethane were distilled over CaH_2_ prior to use. All reactions were performed under an atmosphere of dry nitrogen. Melting points were determined using the capillary method and are uncorrected. Column chromatography employed silica gel (Merck, 130–270 mesh). Thin-layer chromatography (TLC) was performed using precoated silica gel plates (Merck Kieselgel 60F_254_). ^1^H-NMR and ^13^C-NMR spectra were recorded on a JEOL ECA 600 MHz spectrometer (at 600 and 150 MHz, rexpectively) using deuterated chloroform (CDCl_3_) as the solvent, and all chemical shifts are shown in parts per million (ppm) with tetramethylsilane as the internal standard. Mass spectra were measured using a JEOL GCmate mass spectrometer. Elemental analyses were carried out using an elemental analyzer (Choice Analytical Elementar VARIO EL). UV-Vis spectra were recorded on a JASCO V-550 spectrophotometer, and photoluminescence spectra were measured with a JASCO FP-6300 fluorospectrophotometer in cuvettes (in solution) or in vacuum evaporation films on quartz substrates. Cyclic voltammetry was performed using a potentio-stat (Hokuto Denko HZ-5000), and measurement was carried out in dichloromethane containing 0.1 M tetrabutylammonium hexafluorophosphate (Bu_4_NPF_6_) as the supporting electrolyte using a three-electrode cell with a potential scanning rate of 50 mV/s. Suitable single crystals were selected for indexing and intensity data and were measured on a Siemens Smart CCD diffractometer with graphite-monochromated Mo Kα radiation (λ = 0.71073Å) at 293K. The raw data frames were intergrated into SHELX-format reflection files and corrected using the SAINT program. Absorption corrections based on multiscans were obtained by the SADABS program. The structures were solved with direct methods and refined with full-matrix least-squares technique using the SHELXS-97 and SHELXL-97 programs. The coordinates of the non-hydrogen atoms were refined anisotropically, and the positions of the hydrogen atoms were generated geometrically, assigned isotropic thermal parameters, and allowed to ride on their parent carbon atoms until the final cycle of refinement [23,24,25,26].

Crystallographic data have been deposited with the Cambridge Crystallographic Data Centre as CCDC No. 821587, which contains the supplementary crystallographic data for this paper. The data can be obtained free of charge via www.ccdc.cam.ac.uk/conts/retrieving.html (or from the Cambridge Crystallographic Data Centre, 12 Union Road, Cambridge CB2 1EZ, UK; fax (+44) 1223-336-033; e-mail: deposit@ccdc.cam.ac.uk).

### 3.2. Synthesis

*THTP-C*. To a solution of THTP (98 mg, 0.1 mmol) in dry dichloromethane (150 mL) was added a solution of FeCl_3_ (130 mg, 0.8 mmol) in CH_3_NO_2_ (1.5 mL) at 0 °C. After 15 min, anhydrous methanol (30 mL) was added to quench the reaction. The mixture was washed with brine, aqueous saturated NH_4_Cl, and then dried over magnesium sulfate. The residue was purified by column chromatography using hexane/dichloromethane (5:1) as the eluent. After recrystallization by ethyl acetate, a pale yellow solid (76 mg) was obtained at a yield of 78%. mp 185-187 °C. ^1^H-NMR: *δ* 9.48 (s,4H, Ph), 7.58 (s, 4H, Th), 3.11 (t, 8H, *J* = 7.4 Hz, CH_2_), 1.90-1.91 (m, 8H, CH_2_), 1.78 (s, 18H, Bu), 1.50–1.53(m, 8H, CH_2_), 1.35−1.41(m, 16H, CH_2_), 0.92 (t 12H, *J* = 7.2 Hz, CH_3_). ^13^C-NMR: δ 149.0, 147.3, 135.5, 132.9, 128.4, 124.3, 122.5, 122.0, 119.2, 36.0, 32.1, 31.7, 31.3, 30.6, 28.9, 22.6, 14.1. FAB-MS, *m/z*: [M]^+^ 974.3. Anal. Calcd. for C_64_H_78_S_4_: C, 78.79; H, 8.06. Found: C, 78.62; H, 8.17. THTP-C is soluble in common solvents such as chloroform, THF, and even hexane, and can be purified by column chromatography and recrystallization. Considering that TTP has a similar structure to THTP, the same procedures as for THTP-C were used to prepare the other thiophene-fused polycyclic aromatics (TTP-C). However, attempts to perform oxidative cyclodehydrogenation of TTP gave a complicated mixture rather than TTP-C even with a small amount of FeCl_3_ (1.8 equiv. per formed C-C bond) and using diluted reaction concentrations.

## 4. Conclusions

FeCl_3_-mediated oxidative cyclization was successfully used to construct an extended thiophene-pendant pyrene skeleton via thienyl-thienyl carbon-carbon bond formation of 4,5,9,10-tetra[2-(5-hexyl)thienyl]-2,7-di-*tert*-butylpyrene (THTP) and obtain novel thiophene-fused polycyclic aromatic THTP-C with a larger rigid tetracene core, while it could not be successfully applied to electron-rich precursor 4,5,9,10-tetra(2-thienyl)-2,7-di-*tert*-butylpyrene (TTP). The new compound, THTP-C, with a large π-conjugated structure has the tendency to self-associate through intermolecular π-π stacking, which was evidenced by its concentration-dependent ^1^H-NMR spectra in CDCl_3_ and the comparison of its photophysical properties in solution and in the solid state. The optical band gap of THTP-C determined from the absorption onset was found to be 2.86 eV, which is significantly higher than that of traditional pentacene derivatives, which implies a greater stability against photo-degradation. The HOMO energy level of THTP-C determined from the onset oxidation potentials in the cyclic voltammogram was calculated to be −5.64 eV, which is significantly lower than that of traditional p-type semiconductor materials and a closer match with the work function of platinum; this clearly encourages the fabrication of oxidatively stable organic field effect transistor devices based on this material. Crystallographic analyses revealed that THTP-C is not planar but has a curvature similar to “saddle” shaped systems. Moreover, intermolecular π-π and CH–S weak interactions lead to the formation of the resulting 1-D supramolecular structure; such a quasi face-to-face interaction structure is desirable for fast charge transport along the stacking axis and thus THTP-C could potentially serves as semiconductor material for electronic device applications. However, further studies to fabricate THTP-C-based organic field effect transistors and organic light emitting diodes are under way.

## References

[1] Murphy A.R., Fréchet J.M.J. (2007). Organic semiconducting oligomers for use in thin film transistors. Chem. Rev..

[2] Mishra A., Ma C.Q., Bäuerle P. (2009). Functional oligothiophenes: Molecular design for multidimensional nanoarchitectures and their applications. Chem. Rev..

[3] Didane Y., Mehl G.H., Kumagai A., Yoshimoto N., Videlot-Ackermann C., Brisset H. (2008). A “Kite” shaped styryl end-capped benzo[2,1-b:3,4-b’]dithiophene with high electrical performances in organic thin film transistors. J. Am. Chem. Soc..

[4] Kim H.S., Kim Y.H., Kim T.H., Nob Y.Y., Pyo S., Yi M.H., Kim D.Y., Kwon S.K. (2007). Synthesis and studies on 2-hexylthieno[3,2-b]thiophene end-cappedoligomers for OTFTs. Chem. Mater..

[5] Meng H., Sun F.P., Goldfinger M.B., Jaycox G.D., Li Z.G., Marshall W.J., Blackman G.S. (2005). High-performance, stable organic thin-film field-effect transistors based on bis-5’-alkylthiophen-2’-yl-2,6-anthracen semiconductors. J. Am. Chem. Soc..

[6] Merlo J.A., Newman C.R., Gerlach C.P., Kelley T.W., Muyres D.V., Fritz S.E., Toney M.F., Frisbie C.D. (2005). *p*-Channel organic semiconductors based on hybrid acene-thiophene molecules for thin-film transistor applications. J. Am. Chem. Soc..

[7] Noh Y.Y., Azumi R., Goto M., Jung B.J., Lim E.H., Shim H.K., Yoshida Y., Yase K., Kim D.Y. (2005). Organic field effect transistors based on biphenyl, fluorene end-capped fused bithiophene oligomers. Chem. Mater..

[8] Zhang H.J., Wang Y., Shao K.Z., Liu Y.Q., Chen S.Y., Qiu W.F., Sun X.B., Qi T., Ma Y.Q., Yu G. (2006). Novel butterfly pyrene-based organic semiconductors for field effect transistors. Chem. Commun..

[9] Lucas L.A., DeLongchamp D.M., Richter L.J., Kline R.J., Fischer D.A., Kaafarani B.R., Jabbour G.E. (2008). Thin film microstructure of a solution processable pyrene-based organic semiconductor. Chem. Mater..

[10] Sonar P., Soh M.S., Cheng Y.H., Henssler J.T., Sellinger A. (2010). 1,3,6,8-Tetrasubstituted pyrenes: Solution-processable materials for application in organic electronics. Org. Lett..

[11] Duan Z.F., Hoshino D., Yang Z.G., Yano D., Ueki H., Liu Y.W., Ohuchi H., Takyanagi Y., Zhao G.Y., Nishioka Y. (2011). Synthesis and characterization of novel pyrene derivatives containing thienyl groups. Mol. Cryst. Liq. Cryst..

[12] Tovar J.D., Rose A., Swager T.M. (2002). Functionalizable polycyclic aromatics through oxidative cyclization of pendant thiophenes. J. Am. Chem. Soc..

[13] Brusso J.L., Hirst O.D., Dadvand A., Ganesan S., Cicoira F., Robertson C.M., Oakley R.T., Rosei F., Perepichkat D.F. (2008). Two-dimensional structural motif in thienoacene semiconductors: Synthesis, structure, and properties of tetrathienoanthracene isomers. Chem. Mater..

[14] Pei J., Zhang W.Y., Mao J., Zhou X.H. (2006). Helical polycyclic aromatics containing thiophenes: Synthesis and properties. Tetrahedron Lett..

[15] Luo J., Zhao B.M., Chan H.S.O., Chi C.Y. (2010). Synthesis, physical properties and self-assembly of star-shaped oligothiophenes-substituted and fused triphenylenes. J. Mater. Chem..

[16] Zhou Y., Liu W.J., Ma Y.G., Wang H.L., Qi L.M., Cao Y., Wang J., Pei J. (2007). Single microwire transistors of oligoarenes by direct solution process. J. Am. Chem. Soc..

[17] Liu J.Y., Zhang R., Sauve G., Kowalewski T., McCullough R.H. (2008). Highly disordered polymer field effect transistors: *N*-Alkyl dithieno[3,2-b:2’,3’-d]pyrrole-based copolymers with surprisingly high charge carrier mobilities. J. Am. Chem. Soc..

[18] Tang M.L., Reichardt A.D., Wei P., Bao Z.N. (2009). Correlating carrier type with frontier molecular orbital energy levels in organic thin film transistors of functionalized acene derivatives. J. Am. Chem. Soc..

[19] Porzio W., Destri S., Giovanella U., Pasini M., Marin L., Iosip M.D., Campione M. (2007). Solid state properties of oligomers, containing dithienothiophene or fluorene residues suitable for field effect transistor devices. Thin Solid Films.

[20] Wu G.L., Zhao G.J., He C., Zhang J., He Q.G., Chen X.M., Li Y.F. (2009). Synthesis and photovoltaic properties of a star-shaped molecule with triphenylamine as core and benzo[1,2,5]thiadiazol vinylene as arms. Sol. Energ. Mat. Sol. C..

[21] Filatov A.S., Jackson E.A., Scott L.T., Petrukhina M.A. (2009). Foregoing rigidity to achieve greater intimacy. Angew. Chem. Int. Ed..

[22] Anthony J.E., Eaton D.L., Parkin S.R. (2002). A road map to stable, soluble, easily crystallized pentacene derivative. Org. Lett..

[23] Eddaoudi M., Moler D.B., Li H.L., Chen B.L., Reineke T.M., O’Keeffe M., Yaghi O.M. (2001). Modular chemistry: Secondary building units as a basis for the design of highly porous and robust metal-organic carboxylate frameworks. Acc. Chem. Res..

[24] Sun D.F., Collins D.J., Ke Y.X., Zuo J.L., Zhou H.C. (2006). Construction of open metal-organic frameworks based on predesigned carboxylate isomers: From achiral to chiral nets. Chem. Eur. J..

[25] Ma S.Q., Eckert J., Forster P.M., Yoon J.W., Hwang Y.K., Chang J.S., Collier C.D., Parise J.B., Zhou H.C. (2008). Further investigation of the effect of framework catenation on hydrogen uptake in metal-organic frameworks. J. Am. Chem. Soc..

[26] Lin X., Telepeni I., Blake A.J., Dailly A., Brown C.M., Simmons J.M., Zoppi M., Walker G.S., Thomas K.M., Mays T.J. (2009). High capacity hydrogen adsorption in Cu(II) tetracarboxylate framework materials: The role of pore size, ligand functionalization, and exposed metal sites. J. Am. Chem. Soc..

